# White Vortex Light Generations With 7OCB Spherulite

**DOI:** 10.1002/nap2.70032

**Published:** 2026-02-21

**Authors:** Yuanfeng Liu, Le Zhou, Xiaoxuan Peng, Yongzheng Wen, Jingbo Sun, Ji Zhou

**Affiliations:** ^1^ School of Materials Science and Engineering, State Key Laboratory of New Ceramic Materials Tsinghua University Beijing China; ^2^ Department of Physics Colorado School of Mines Golden Colorado USA

**Keywords:** 7OCB, optical vortex, spherulite, white light

## Abstract

A spherulite is a radially symmetric, microscale crystalline aggregate formed by molecular self‐assembly, typically exhibiting concentric birefringent textures under polarized light, which is highly sought after for optical applications, especially in structured light generation and modulation. In this work, we exploit the optical properties of spherulites formed by 7OCB molecules. Radially aggregated needle‐like 7OCB crystals result in a strong anisotropic transmittance with respect to radial and azimuthal orientations due to scattering loss over a wide range. Thus, polychromatic generation of cylindrical vector optical vortex beams across a broad spectrum from visible to near infrared, as well as a noncoherent white light optical vortex beam, is realized via spin‐to‐orbital angular momentum conversion from the spherulite. This approach opens promising opportunities for employing spherulites in structured‐light generation and in the modulation of both polarization and angular momentum.

## Graphical abstract

A spherulite made of radially arranged needle‐like 7OCB crystals exhibits a high contrast regarding polarized transmittance along radial and azimuthal directions across the entire visible range. Thus, it can function as an efficient generator of broadband polychromatic optical vortices, as well as a noncoherent white light vortex beam.

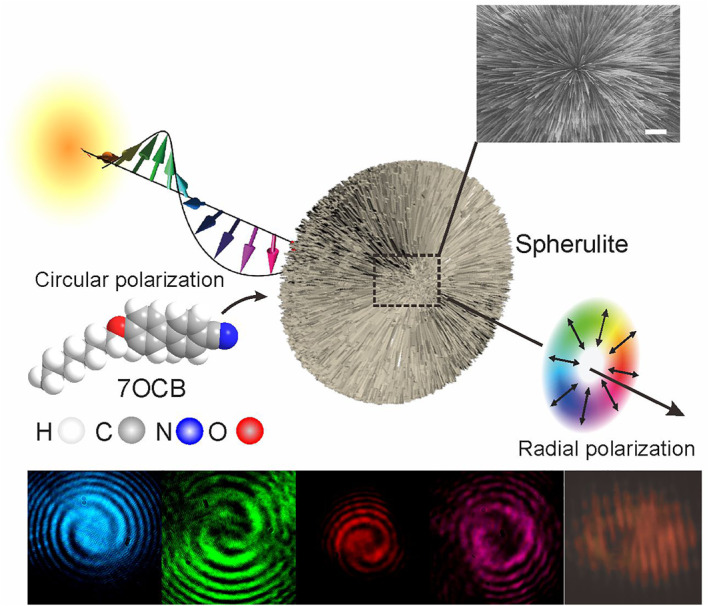

## 1 Introduction

Optical vortices, characterized by helical phase fronts and the ability to carry orbital angular momentum (OAM), have emerged as a central resource in modern photonics [1, 2, 3, 4, 5]. Their unique properties underpin a wide range of applications, from high‐capacity optical communications [6, 7, 8, 9, 10] and quantum information processing [11, 12] to super‐resolution imaging [13, 14], optical trapping [15, 16], and micromanipulation [17, 18]. A variety of methods have been developed for vortex generation, including spiral phase plates [18, 19, 20], q‐plates [21, 22], spatial light modulators [23, 24], and metasurfaces [25, 26, 27, 28, 29, 30]. Among these, metasurfaces have attracted particular attention for their ability to achieve precise subwavelength control of the phase, amplitude, and polarization of light within compact planar architectures, making the dynamic tuning and multifunctional integration possible [25, 27, 31, 32, 33, 34].

Despite these advances, metasurface‐based approaches face intrinsic limitations. Almost all metasurfaces require sophisticated top‐down nanofabrication, which is costly, time‐consuming, and difficult to scale to large‐area production [26, 27, 32, 33, 34]. In addition, material absorption and scattering loss become significant challenges, particularly in the visible range [35], where many photonic applications are most demanding. These limitations motivate the exploration of alternative strategies for vortex beam generation that can combine fabrication simplicity, broadband performance, and scalability.

Spherulites, radially symmetric crystalline aggregates formed via molecular self‐assembly, present a naturally endowed platform that addresses these challenges [36, 37, 38]. Unlike engineered nanostructures, spherulites intrinsically exhibit circular anisotropy that is inherently matched to the cylindrical symmetry of structured light [39, 40, 41, 42, 43, 44, 45, 46, 47]. This property enables direct spin‐to‐orbital angular momentum conversion across a broad visible range, without the need for nanofabrications. As a bottom‐up, self‐assembled material system, spherulites offer a low‐cost, scalable, and broadband solution for vortex beam generation while also allowing modulation of both polarization and angular momentum.

In this work, we demonstrate white light vortex generation from a spherulite composed of radially arranged needle‐like crystals of 7OCB molecules, which exhibits high loss with respect to azimuthal polarization in the visible–near‐infrared range. Incident beams with circular polarization can be transformed into radially polarized vortex beams through spin‐to‐orbital angular momentum conversion. The broadband circular anisotropy not only enables polychromatic vortex generations for lasers but can also be used to achieve noncoherent white vortex light. This approach highlights the unique photonic potential of molecularly self‐assembled crystalline structures, providing a disruptive alternative to metasurface‐based devices and opening new opportunities for structured‐light applications.

## 2 Results

### 2.1 Design

**FIGURE 1:**
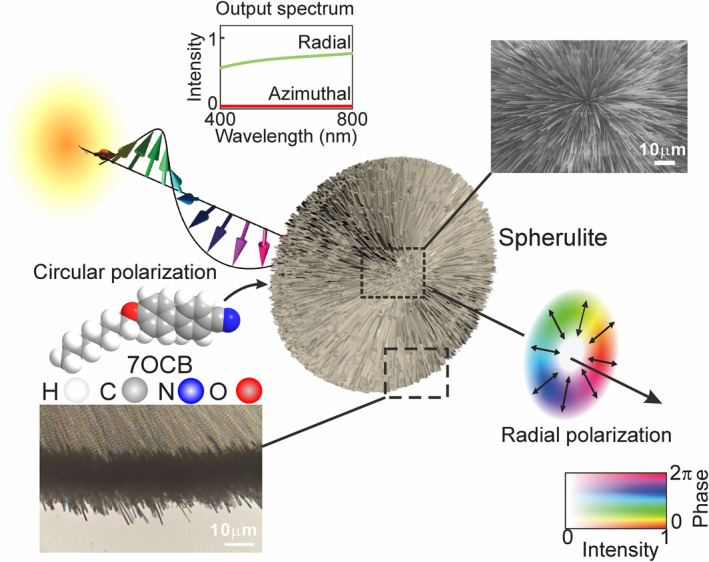
Schematic of the generation of a vector vortex beam using a dichroic scattering spherulite. The radially distributed 7OCB crystals may result in strong anisotropy in transmission with respect to radial and azimuthal polarizations. Thus, the incident beam with circular polarization is converted into a radially polarized optical vortex through spin‐orbital angular momentum coupling. The scanning electron microscope and optical microscope images show the center and the edge of the spherulite, respectively.

The spherulite materials are fabricated through crystallization of 7OCB molecules by a quenching process. The preparation of the spherulites can be found in Supporting Information S1. A large amount of spherulites can be obtained in one crystallization; see Supporting Information S1: Figure S2. As shown in Figure 1, the spherulite is formed by needle‐like 7OCB crystals, aggregated with the axis of each needle aligned along the radial direction. Scanning electron microscope and optical microscope images (inset in Figure 1) show that the diameters of the crystalline needles are approximately 500 nm, and the longitudinal size can be as long as several tens of the micrometers. Intrinsically, the 7OCB material is transparent with respect to light. Because of geometric anisotropy, the array of needles may show optical anisotropy. If the *E* field of the incident light beam is perpendicular to the needles, strong scattering can occur for light with wavelengths in the visible or near‐infrared range because of the needle diameters. However, light beams polarized along the longitudinal direction can transmit through the needle array with relatively low loss. Once the needle‐like crystal aggregates in a radial way, a circular anisotropy is achieved, with high transmittance for polarization along the radial direction and low transmittance along the azimuthal direction, which can be used to convert a circularly polarized beam into a radially polarized optical vortex through spin‐orbital angular momentum coupling.

### 2.2 Optical Characterizations on Spherulites

**FIGURE 2:**
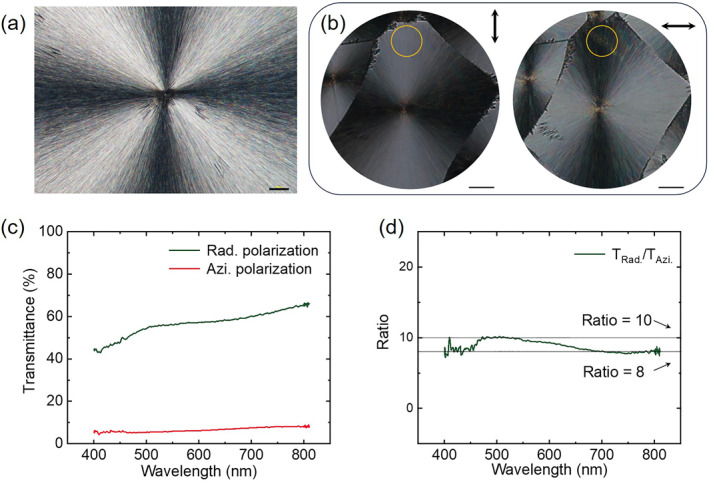
Optical characterizations of the anisotropy of the spherulites. (a) Optical image under a polarized optical microscope, which shows the Maltese cross. The scale bar is 100 μm. (b) Microregion used for transmittance measurement. The scale bar is 50 μm. (c) Transmittance spectra of radial (*T*_Rad_) and azimuthal (*T*_Azi_) polarizations. (d) The contrast of the transmittance in the visible range. Azi, azimuthal; Rad, radial.

Such optical anisotropy can be demonstrated under a polarized optical microscope. The overall size of a spherulite is on a scale of several hundred micrometers. When the incident beam is a linearly polarized beam and the output of the transmission is filtered by a linear polarizer normal to the incident polarization, we can see a dark cross, which is the Maltese cross, as shown in Figure 2a. Without the linear polarizer, under vertically polarized white light incidence, a bright bow‐tie region appears and is aligned along the vertical direction. When the incident beam is horizontally polarized, the bright bow‐tie region is aligned along the horizontal direction, which implies that the spherulite shows high transmittance along the radial direction, as shown in Figure 2b. To quantitatively describe this anisotropy, we measure the transmittance spectra of orthogonal polarizations along different orientations of the spherulite, which should be performed with the incident beam of radial and azimuthal polarizations in principle. In practice, we achieve the information through a microspectrum system (see Supporting Information S1: Figure S3). As shown in Figure 2b, the incident beam is focused onto a small region (the orange circle) that is far enough from the spherulite's center. In this case, the local area exhibits orthogonal anisotropy approximately corresponding to the conventional Cartesian coordinate system. In our experiment, the microregion is taken right above the center of the spherulite, where the transmittance under horizontal polarization is equivalent to that of azimuthal polarization, whereas the vertical polarization corresponds to radial polarization. The measured anisotropic spectra are shown in Figure 2c, which show high contrast in transmittance over the range from 400 to 810 nm, as indicated by *T*_Rad_/*T*_Azi_, as shown in Figure 2d. For the anisotropic reflectance, no pronounced reflection dichroism is observed (see Supporting Information S1: Figure S4), indicating that scattering contributes minimally to the overall reflection.

Such a circularly anisotropic transmittance property can be used to generate optical vortex beams through spin‐orbital angular momentum coupling over a wide range from visible to near infrared. A circularly polarized beam with spin angular momentum can be considered a linear combination of a radially polarized optical vortex and an azimuthally polarized optical vortex with a topological charge of *l* = ± 1, with a phase difference of π/2 between them. The left and right circular polarizations are expressed as follows [48, 49, 50]:

(1a) $$E_{L}=\frac{P\left(r\right)\left(e_{r}+ie_{\theta }\right)}{\sqrt{2}}e^{i\theta },$$

and

(1b) $$E_{R}=\frac{P\left(r\right)\left(e_{r}-ie_{\theta }\right)}{\sqrt{2}}e^{-i\theta },$$

respectively. In Equations (1a) and (1b), *P*(*r*) is the amplitude distribution of the beam in the cylindrical coordinate, and ***e***_***r***_ and ***e***_***θ***_ are the unit vectors along the radial and azimuthal directions, respectively. *θ* is the azimuthal angle. Through this coordinate transformation, the original spin angular momentum is still there but hidden in the radial and azimuthal vectors (***e***_***r***_ ± *i* ***e***_***θ***_), which carry the OAM, providing an approach for generating optical vortices of cylindrical vector beams.

### 2.3 Optical Vortex Generations

According to the theory described above, separation of the radial and azimuthal components can transform the spin angular momentum into OAM permanently. When a circularly polarized beam is incident onto the spherulite, the azimuthal component is filtered out due to strong scattering, and only the radial component can pass through, resulting in an output beam with radial polarization as well as OAM of a topological charge of *l* = ± 1. Because the highly anisotropic transmittance exists across the entire visible range, we can obtain not only the polychromatic coherent optical vortex beams but also a white light optical vortex beam.

**FIGURE 3:**
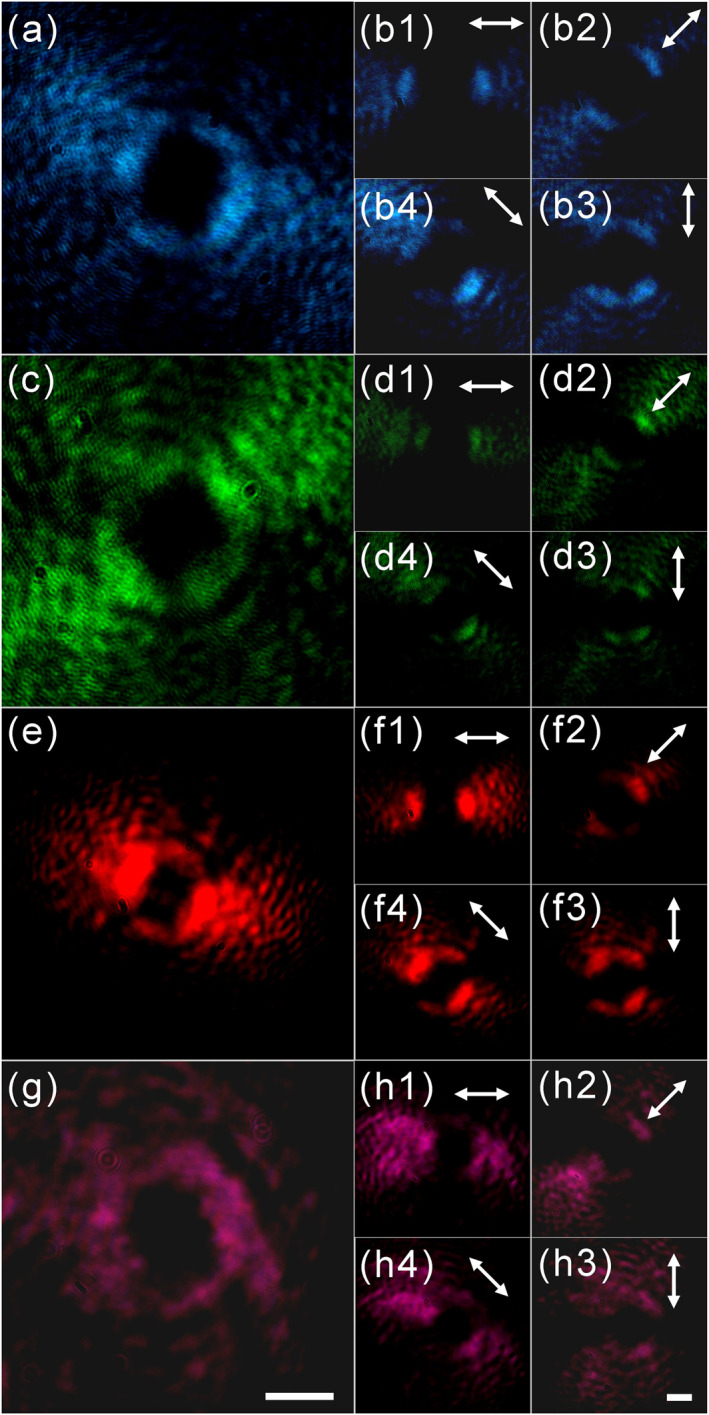
Polarization states of the output beams. (a, c, e, and g) Output beam's intensity profiles at 488, 532, 633, and 808 nm. (b, d, f, and h) Intensity profiles after a linear polarizer at angles of 0^ο^, 45^ο^, 90^ο^, and 135^ο^ for each wavelength. Panels (a, c, e, and g) share the same scale bar shown in Panel (g), which is 500 μm. Panels (b, d, f, and h) share the same scale bar shown in Panel (h), which is 500 μm.

In the experiment, linearly polarized continuous‐wave lasers at wavelengths of 488 (OBIS 488‐20LS), 532 (Coherent Verdi V6), 633 (GY‐11[DH‐HN250]), and 808 (OBIS 808LX) nm are first converted into right circular polarization through an achromatic quarter‐wave plate and then focused onto the sample. The focused beam size is smaller than the size of one single spherulite, and the light beams' center is coaxial with the center of the spherulite. After transmission through the spherulite, beams at all these four wavelengths show the donut shape in the intensity profile, as shown in Figure 3a,c,e,g. The central singularity in the intensity profile is due to high loss at the center of the spherulite. The spherulite shows circularly anisotropic scattering overall. In a local region that is far from the center, only the azimuthally polarized beam is seriously scattered. However, close to the central region, it is equal to an isotropic scattering region that may attenuate beams of all polarizations.

The polarization states of the output beam are tested using a linear polarizer. As shown in Figure 3b,d,f,h, the donut‐shaped intensity profile of the output beam splits into two lobes aligned with the orientation of the polarizer. When we rotate the polarizer along different angles: 0^ο^, 45^ο^, 90^ο^, and 135^ο^, the two‐lobe pattern follows the polarizer's rotation, which demonstrates that the azimuthally polarized component in each incident beam has been fully removed, and thus, the generated beams are purely radially polarized for all four wavelengths.

According to Equations (1a) and (1b), when the azimuthal polarization is filtered out, OAM is transformed from the spin angular momentum of the incident beam, and the generated beam carries OAM with a topological charge *l* = −1, which can be verified through the interference.

The topological charge of the output beam is explored through a superposition of two orthogonal circular polarizations. Based on Equations (1a) and (1b), a radially polarized vortex beam with a topological charge of *l* = −1 is comprised of right and left circularly polarized beams with charge *l* = 0 and charge *l* = −2, respectively:

(2) $$E_{r}e^{-i\theta }=P\left(r\right)e_{r}e^{-i\theta }=\frac{E_{R}+E_{L}e^{-i2\theta }}{\sqrt{2}}.$$

**FIGURE 4:**
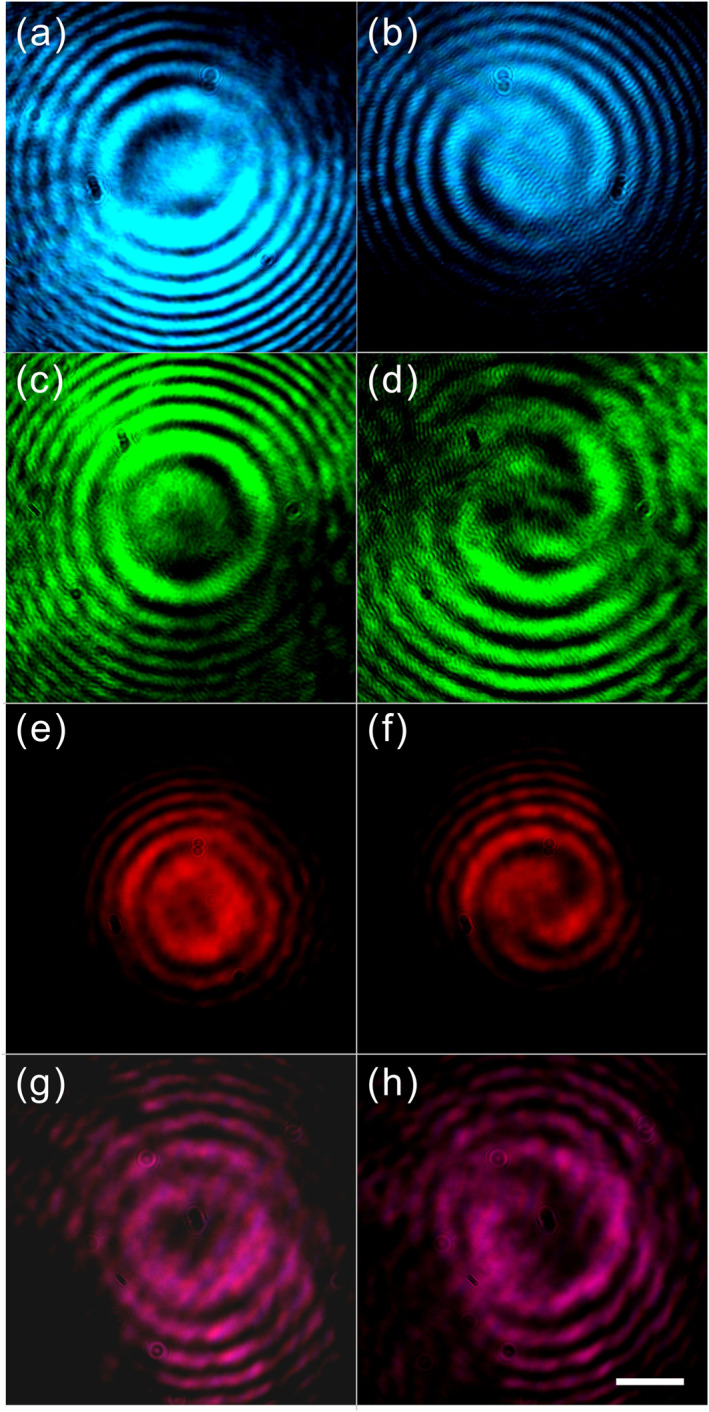
Interference patterns formed by the generated beams and the reference beams of opposite spins at four wavelengths. (a, c, e, and g) Ring interference patterns obtained by using right circular polarization at 488, 532, 633, and 808 nm, respectively, which indicate that no OAM exists in the right circular components. (b, d, f, and h) Interference patterns obtained by using left circular polarization at 488, 532, 633, and 808 nm, respectively, all of which are twofold spiral patterns, implying the OAM with a topological charge of *l* = −2 in the left circular polarization component. The scale bar is 500 μm, applied to all panels.

Therefore, in the experiment, we use a Mach–Zehnder setup (see Supporting Information S1: Figure S3) with reference beams of two opposite spin directions to interfere with the generated beams at each wavelength, and the results are shown in Figure 4. When the reference beams are in right circular polarization, the same as the incident polarization, the interference patterns show only concentric rings and thus imply no OAM, as shown in Figure 4a,c,e,g, whereas in the case of left circular polarization, twofold spiral interference patterns appear at all four wavelengths, as shown in Figure 4b,d,f,h, showing the existing OAM with a topological charge of *l* = −2 in the left circularly polarized component. Such results manifest that the output beams carry OAM with a topological charge of *l* = −1 in radial polarization, according to Equation (2). The purity of the generated vortex beams is characterized in Supporting Information S1: Text 3.

**FIGURE 5:**
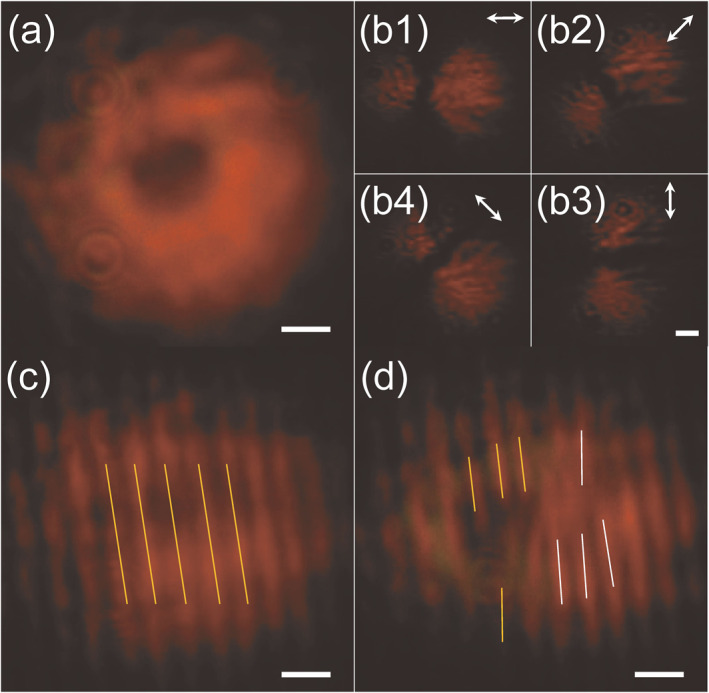
White light vortex generated by the spherulite. (a) Output intensity profile. (b) Intensity profile after a linear polarizer at angles of 0^ο^, 45^ο^, 90^ο^, and 135^ο^. (c) Self‐interference pattern of the right circular component. (d) Self‐interference patterns of the left circular component. The scale bar is 200 μm. The incident polarization is in the right circular state. The overall color of the noncoherent light shows a strong red–orange hue because of the spectral sensitivity of the camera. Further spectral characterization of the input/output beam indicates the noncoherent white light feature, as shown in Supporting Information S1: Figure S8.

Based on the results of coherent polychromatic vortex beam generation, we explore the possibility of generating a white light optical vortex by using the spherulite. A noncoherent white light beam from a stabilized tungsten–halogen light source is first collimated by a plano‐convex lens. A linear polarizer and a superachromatic quarter‐wave plate covering the wavelength range of 325–1100 nm are used to transform the incident white light into circular polarization. Then, it is focused onto a spherulite with a lens. At the output side, we obtain a white‐colored donut‐shaped pattern, as shown in Figure 5a. Similarly, by applying a linear polarizer to analyze the intensity profiles, we can also conclude that the output white light beam is in radial polarization, according to Figure 5b, and thus deduce the existence of the OAM with a topological charge of *l* = −1, which is further experimentally confirmed by interference analysis, as shown in Figure 5c,d.

In the experiment, we use a self‐interference configuration (see Supporting Information S1: Figure S7), where the right or left circular polarization component of the generated white light beam interferes with itself. For the right circular polarization component, the interference patterns show only parallel fringes and thus imply no OAM, as shown in Figure 5c, whereas in the case of the left circular polarization component, fork‐like interference fringes appear, as shown in Figure 5d, showing the existing OAM with a topological charge of *l* = −2 in the left circularly polarized component. Such results manifest that the output white light beam carries OAM with a topological charge of *l* = −1 in radial polarization, according to Equation (2). The achromatic generation of wideband optical vortices may empower the modulation of optical spatiotemporal vortices [51], wavelength division multiplexing of OAM [52], and novel light–matter interaction phenomena under broad operating bandwidths [53, 54].

## 3 Conclusions

In this work, polychromatic visible optical vortex beams are experimentally realized using a 7OCB‐based spherulite material. Radially distributed nano‐needle‐like 7OCB crystals cause an achromatic cylindrically anisotropic transmittance, which converts a circularly polarized incident beam into a radially polarized vortex beam across the entire visible range. Unlike metamaterials or metasurfaces with artificially engineered cylindrical symmetry, the spherulite of a large scale can be fabricated through simple condensational crystallization. The intrinsic cylindrical anisotropy of spherulites offers a promising new pathway for exploring novel light–matter interaction phenomena with a wide working bandwidth.

## Author Contributions

**Yuanfeng Liu:** conceptualization, methodology, formal analysis, writing – review and editing, funding acquisition. **Le Zhou:** formal analysis, review and editing. **Xiaoxuan Peng:** formal analysis. **Yongzheng Wen:** validation. **Jingbo Sun:** conceptualization, methodology, investigation, validation, formal analysis, supervision, funding acquisition, writing – original draft, writing – review and editing. **Ji Zhou:** supervision, funding acquisition, review and editing.

## Funding

This work was supported by the National Key R&D Program of China (Grant No. 2022YFB3806000), NSFC (1257041442), the NSFC Key Program (Grant No. 52332006), and the Postdoctoral Fellowship Program of CPSF under Grant No. GZC20250073.

## Conflicts of Interest

The authors declare no conflicts of interest.

## Supporting information


Supporting Information S1

## Data Availability

The datasets generated and/or analyzed during this study are available from the corresponding author upon reasonable request.
