# Supplementary material for: White Vortex Light Generations With 7OCB Spherulite
Source: Nanophotonics. 2026 Feb 21;15(4):e70032. doi: 10.1002/nap2.70032 (PMC12965004; doi:10.1002/nap2.70032)
Supplement: Supplementary file 1 — Supporting Information S1 [file NAP2-15-e70032-s001.pdf]

Supplementary information for

White vortex light generations with 7OCB spherulite

Yuanfeng Liu, Le Zhou, Xiaoxuan Peng, Yongzheng Wen, Jingbo Sun* and Ji Zhou*

Yuanfeng Liu, Xiaoxuan Peng, Yongzheng Wen, Jingbo Sun, and Ji Zhou: School of Materials Science and Engineering, State Key Laboratory of New Ceramic Materials, Tsinghua University, Beijing, China

Le Zhou: Department of Physics, Colorado School of Mines, Illinois, St, Golden, USA

* To whom correspondence may be addressed. Email: jingbosun@tsinghua.edu.cn (J. Sun), zhouji@tsinghua.edu.cn (J. Zhou).

Y. Liu and L. Zhou contributed equally to this work.

Supplementary Text 1. Sample preparation

Differential scanning calorimetry test. Before preparing the spherulite crystals, we need to firstly know about the phase transformation temperature of the as-used 7OCB, by using a differential scanning calorimeter (DSC Q2000, TA Instruments). The 7OCB raw material (purity: 98%) was purchased from Aladdin. In the heating and cooling process, an endothermic or exothermic peak appeared at ~74.5 °C, corresponding to the transition between nematic phase and isotropic phase [1].

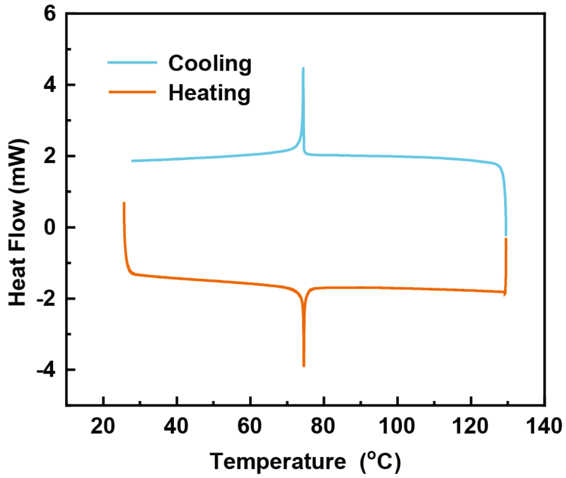

Figure S1. DSC profile of the 7OCB powder material (3.26 mg) in a heating-cooling process at the rate of 10 °C min^{-1}.

Preparation of 7OCB spherulite. Based on the thermal analysis results provided in Figure S1, approximately 2 mg of the material was melted at 130 °C for 3 min on a glass substrate to eliminate its thermal history. The molten 7OCB was then covered with a glass slide to form a sandwich-like configuration. Rapid cooling was achieved by placing the sample on a flat metal heat sink, inducing the formation of 7OCB spherulites through molecular self-assembly.

Therefore, compared to the nano-fabrication methods, such as focused ion beam etching or e-beam lithography together with dry etching, this spherulite crystal growing method is much easier and faster. Meanwhile, the production of the

spherulites is also considerable. After quenching, there are a huge amount of the spherulites, close packed on the glass substrate. Figure S2 shows just a small part of the optical image from one piece of the glass slide sample. These spherulites exhibit a high degree of structural similarity, suggesting favorable fabrication consistency and efficiency. The spherulites show good structural stability below its phase transition temperature.

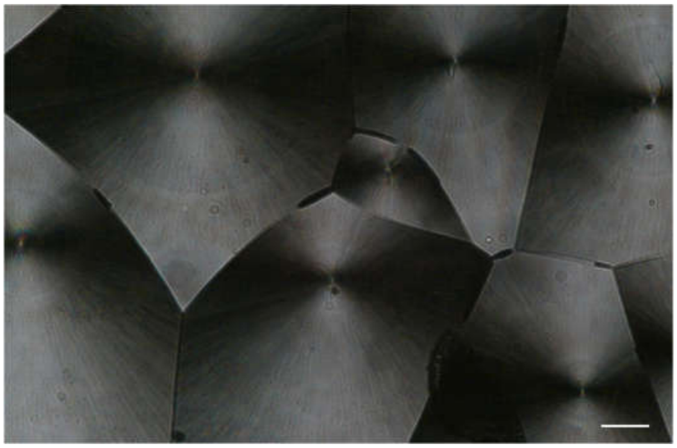

Figure S2. Optical polarizing microscope image of multiple 7OCB spherulites under transmission mode. The incidence is in vertical-linear polarization. The scalebar is 100 μm.

Microstructure characterization. The spherulite surface was characterized using a scanning electron microscope (Merlin, Carl Zeiss). Prior to observation, the sample was coated with a 20 nm-thick layer of platinum (Pt). The SEM was operated at an accelerating voltage of 2 kV to minimize electron beam–induced damage. The optical polarizing response of the spherulite was analyzed using an optical microscope (Ti-E, Nikon) for polarized light imaging.

Supplementary Text 2. Optical characterizations

General setup. The setup for the micro-region spectrum acquisition and structured beam characterizations can be found in Figure S3. The input 1 is used for the coupling of white light. The iris before the camera and the optical fiber is used to confine the

micro-region for imaging or spectra acquisition. The input 2 is used for the coupling of lasers with different wavelengths for the Mach-Zehnder-type optical configuration. The incident polarization is modulated by a pair of polarizer and waveplate.

Transmittance spectrum measurement. The anisotropic transmittance of the spherulite was measured using a micro-area optical testing system. A mercury-xenon lamp (SLS401, Thorlabs) was used as the light source to provide a broad spectral range spanning 240 to 2400 nm. The resulting spectra were acquired using a spectrometer: HR2000+ES from Ocean Optics. Measurements were performed on a micro-region located away from the spherulite center. Using linearly polarized light oriented along the radial and azimuthal directions, the transmittance anisotropy spectra were obtained.

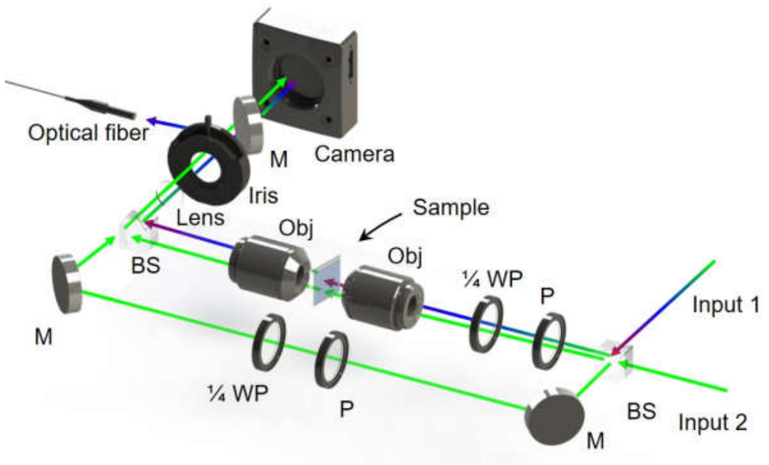

Figure S3. Setup for the optical characterizations. M: mirror, P: polarizer, WP: waveplate, Obj: objective, BS: beam splitter.

Reflectance spectrum measurement. The anisotropic reflectance of the spherulite was also measured using a micro-area optical testing system in Figure S3. The reflected beam was captured using an objective (10×, NA 0.25, Olympus). Similar to the transmittance spectra acquisition, the measurements were performed on a micro-region located away from the spherulite center. Using linearly polarized light oriented along the radial and azimuthal directions, the reflection anisotropy spectra were

obtained. The reflection spectra were normalized to the spectra from a silver mirror to obtain the reflectance (see Figure S4). The average reflectance difference of the radial and azimuthal polarizations is 1.6 %.

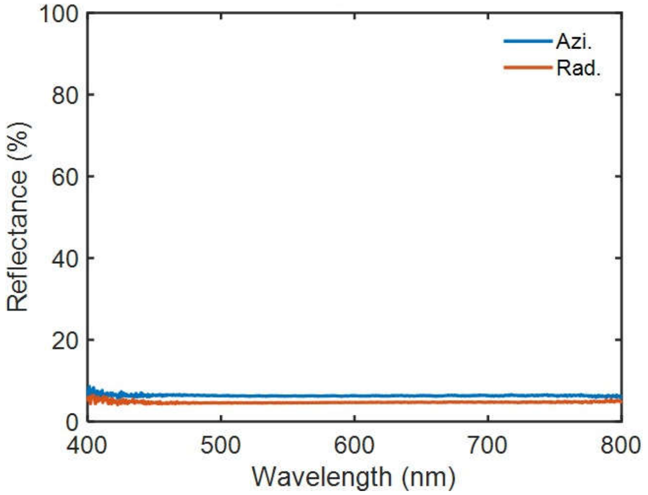

Figure S4. Reflectance of the radial (Rad.) and azimuthal (Azi.) polarizations.

Characterization of generated vector vortex beam. To characterize the broadband optical response of the spherulite across the ultraviolet to near-infrared spectral range, continuous laser sources with wavelengths of 488 nm, 532 nm, 633 nm, and 808 nm were used. The incident beam was adjusted to generate circularly polarized light. The input beam was slightly focused to match the size of the spherulite under test. The beam center was aligned with the center of the spherulite. A Mach-Zehnder-type optical configuration was employed to perform interference measurements, where the reference beam was set as either right- or left-circularly polarized.

Supplementary Text 3. Purity analysis of the generated radially-polarized vortex

In order to deduce the purity of the phase vortex in the generated beams, we experimentally captured the interference pattern with a plane wave as the reference beam (see Figure S5). When the reference beam is in left circular polarization (LCP) state, the interference pattern shows fork shaped features of the topological charge l=-2. When the reference beam is in right circular polarization (RCP) state, the

interference pattern shows parallel stripe features of the topological charge l=0. The results in Figure S5 are consistent with that in Figure 5 of the main text.

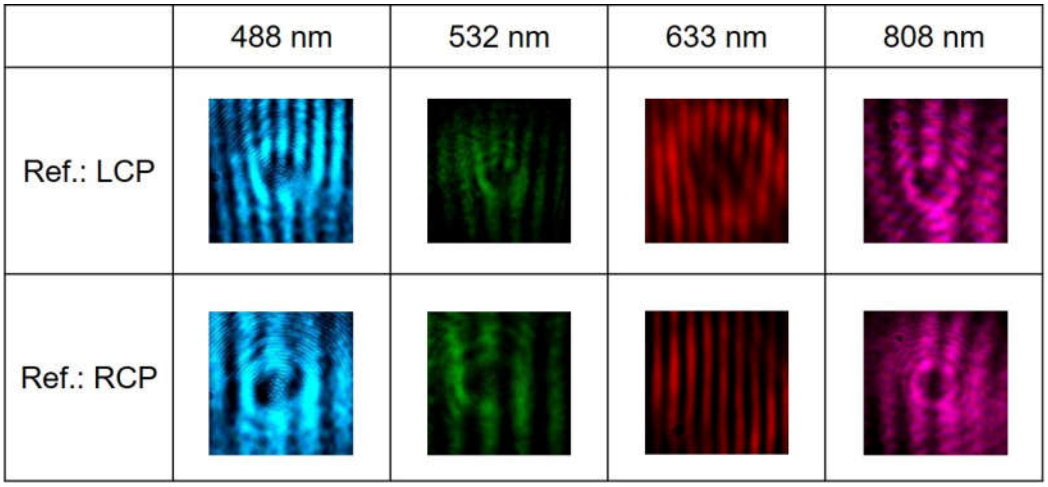

Figure S5. Interference patterns formed by the generated beams and the reference beams of opposite spins at four wavelengths. The reference beam is in plane-wave state with uniform wavefront phase distribution. Ref.: Reference beam.

According to the literature[2], we performed the polarization vortex purity calculations. To deduce the polarization quality of the generated beam, we rotated the polarizer at different angles (see Figure 3), then obtained the intensity distributions $I_{\exp}(r,\theta)$ at four different polarization directions (0°, 45°, 90°, 135°), where r is the radial distance respect to the beam center and θ is the azimuthal angle. The polarization vortex purity was finally deduced by equation S1.

$$P_{purity} = (1-\sum\frac{\left|I_{\exp}(r,\theta)-I_{theo}(r,\theta)\right|}{I_{\exp}(r,\theta)+I_{theo}(r,\theta)})\times 100\% \qquad \text{(S1)}$$

where the I_{theo} is the theoretical intensity of a radial-polarization vortex beam at the give polarization direction and position (r,θ). The calculated average purity of the polarization vortex is at ~76% level, which can be further improved by increasing the sample thickness, i.e., fully eliminating the azimuthal-polarization component in the output. All these results are summarized in Table S1.

Wavelength	488 nm	532 nm	633 nm	808 nm
Phase vortex purity (%)	83.4	80.8	78.8	91.3
Polarization vortex purity (%)	72.1	79.9	77.6	74.4

Table S1. Calculated polarization and phase vortex purities of the generated beams at four wavelengths.

From the interferogram with plane wave as the reference (Figure S5), we extracted the phase distributions of LCP component and RCP component of the generated beam[3], noted as $\varphi_{LCP}(r,\theta)$, and $\varphi_{RCP}(r,\theta)$, respectively. We then deduced the synthetic radial-polarized E-field by the coherent superposition of the LCP and RCP components, as shown in equation (S2).

$$\boldsymbol{E}_{radial} = (\cos\theta + i\sin\theta)\,\mathrm{e}^{i\varphi_{LCP}} + (\cos\theta - i\sin\theta)\,\mathrm{e}^{i\varphi_{RCP}} \qquad \text{(S2)}$$

where θ is the azimuthal angle. Based on the phase distribution of the deduced $\boldsymbol{E}_{radial}$, the purity of the optical phase vortex can be calculated by decomposing its complex field on a basis set of optical vortex mode with topological charge l=±1[3], where the sign depends on the spin of the incidence. The average purity of the phase vortex is at ~84% level. For example, the generated radially-polarized beam at 808 nm clearly shows the phase vortex of the topological charge l=-1 with the purity of 91.3% (see Figure S6).

The experimentally tested polarization and phase vortex purities are comparable with the previous study[2], and could be much improved by optimizing the sample thickness and testing devices.

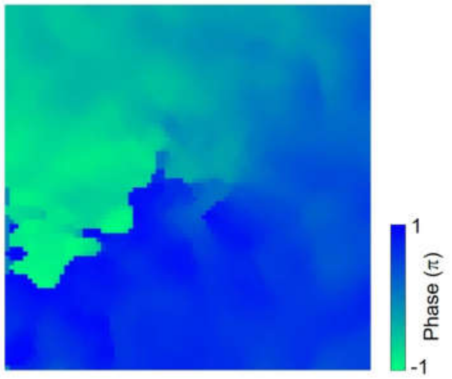

Figure S6. Phase distribution of the experimentally generated radially-polarized optical vortex at 808 nm.

Supplementary Text 4. Interference of the generated white light vortex

The setup of the white light interference is shown in Figure. S7. The incident beam was from a white light source (SLS201L/M, Thorlabs). A pair of polarizer and achromatic quarter waveplate (SAQWP05M-700, Thorlabs) was mounted for the incident polarization modulation. The waveplate works from 325 nm to 1100 nm. The incident beam was focused into a pinhole (diameter: 15 μm) by a 35 mm plano-convex lens. The output beam was collected by a following objective (10×, NA 0.25, Olympus).

The output beam was then focused into the spherulite sample by a 50 mm plano-convex lens. The beam and the spherulite were well aligned. The generated white light vortex was then collected by a following objective (10×, NA 0.25, Olympus).

The output beam was splitted by a non-polarizing beam splitter. The two separated beams were then reflected back into the beam splitter to form a self-interference configuration. Before the camera (MUS500C-G), a 150 mm plano-convex lens was used to image the two white light vortex beams. A pair of achromatic quarter waveplate and polarizer was mounted to extract the left circular or right circular polarization component. The two beams should present the similar optical path length,

which was controlled by a delay line configuration based on a high precision motion stage (DDSM100, Thorlabs).

The spectra of the input white light source and the generated white light vortex were captured by a fiber optic spectrometer (HR2000+ES, Ocean Optics).

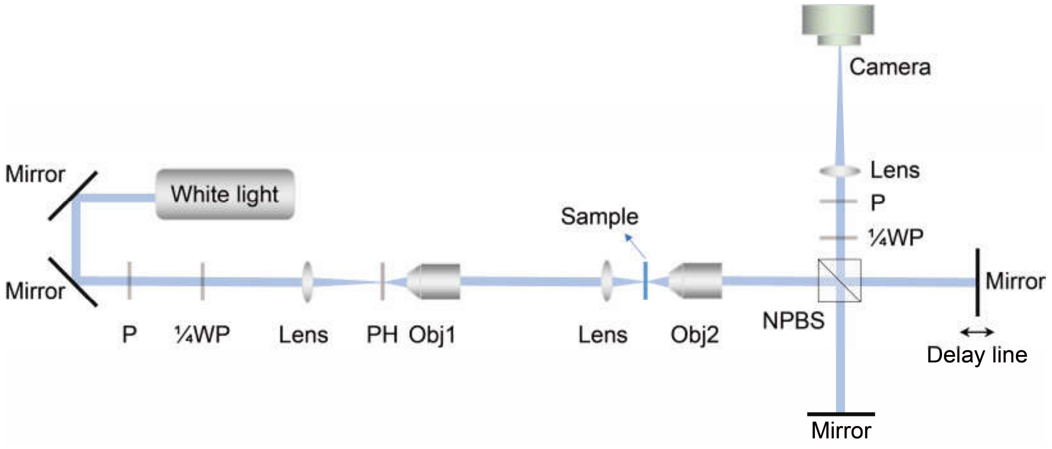

Figure S7. Experimental setup for the interference characterization of the wideband white light vortex. PH: pinhole. P: polarizer. NPBS: non-polarizing beam splitter. Obj: objective. WP: waveplate.

Furthermore, the achromatic capability of the spherulite in generating white light optical vortex was experimentally confirmed. As shown in Figure. S8(a)-(b), the input and output beams show quite similar spectral distributions and close positions in a CIE1931 chromaticity diagram.

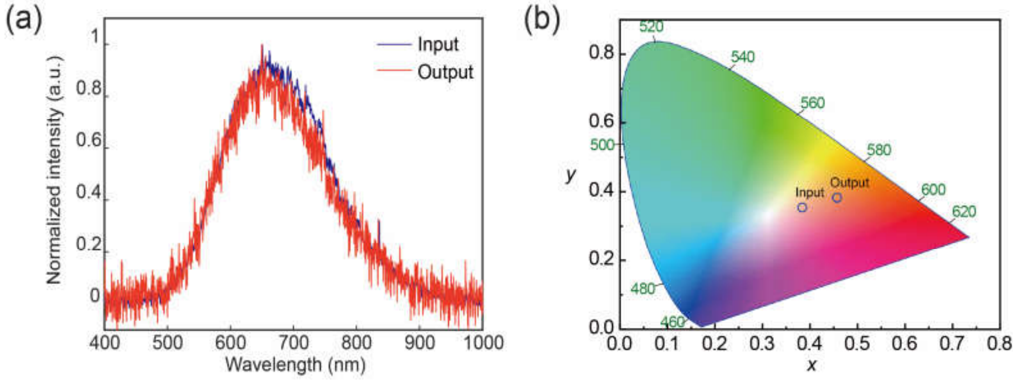

Figure S8. Characterization of the generated wideband white light vortex. (a) Spectra of the input white light source and the generated wideband white light vortex. (b) The positions of the input beam and the generated wideband white light vortex in a CIE1931 chromaticity diagram.

References:

[1] G.A. Oweimreen and M.A. Morsy, "DSC studies on p-(n-alkyl)-p′-cyanobiphenyl (RCB's) and p-(n-alkoxy)-p′-cyanobiphenyl (ROCB's) liquid crystals," *Thermochimica Acta*, vol. 346, no. 1-2, pp. 37-47, 2000.

[2] Y. Hui, et al., "Tunable vector vortex beam vertical cavity surface emitting laser," *Adv. Funct. Mater.*, vol. 35, no. 33, p. 2425931, 2025.

[3] P. Genevet, et al., "Ultra-thin plasmonic optical vortex plate based on phase discontinuities," *Appl. Phys. Lett.*, vol. 100, no. 1, p. 013101, 2012.
